# Supplementary material for: Synergistic effect of tumor chemo-immunotherapy induced by leukocyte-hitchhiking thermal-sensitive micelles
Source: Nat Commun. 2021 Aug 6;12:4755. doi: 10.1038/s41467-021-24902-2 (PMC8346467; doi:10.1038/s41467-021-24902-2)
Supplement: Supplementary file 1 — Supplementary Information [file 41467_2021_24902_MOESM1_ESM.pdf]

Supplementary Information

Synergistic effect of tumor chemo-immunotherapy induced by leukocyte-hitchhiking thermal-sensitive micelles

Jing Qi,[1,#] Feiyang Jin,[1,#] Yuchan You,[1] Yan Du,[1] Di Liu,[1] Xiaoling Xu,[1,*] Jun Wang,[1] Luwen Zhu,[1] Minjiang Chen,[1] Gaofeng Shu,[2] Liming Wu,[3,*] Jiansong Ji [2,*] and Yongzhong Du[1,*]

[1] Institute of Pharmaceutics, College of Pharmaceutical Sciences, Zhejiang University, 866 Yu-Hang-Tang Road, Hangzhou, 310058, China.
[2] Key Laboratory of Imaging Diagnosis and Minimally Invasive Intervention Research, Lishui Hospital of Zhejiang University, Lishui, 323000, China.
[3] Department of Hepatobiliary and Pancreatic Surgery, The First Affiliated Hospital, Zhejiang University School of Medicine, Hangzhou 310003, China.
[#] These authors contributed equally to this work.

[*]Corresponding Authors:
Yongzhong Du, E-mail: duyongzhong@zju.edu.cn, Phone: +86-571-88208435
Xiaoling Xu, E-mail: ziyao1988@zju.edu.cn
Jiansong Ji, E-mail: lschrjjs@163.com, Phone: +86-578-2285011
Liming Wu, E-mail: wlm@zju.edu.cn

Content

Page

Supplementary Fig. 1 Synthesis route of NTA-PEG-p-(AAm-co-AN) and the illustration of the introduction of E-selectin onto the surface of micelles. **S4**

Supplementary Fig. 2 The ^{1}H-NMR spectra of p-(AAm-co-AN), PEG, PEG-p-(AAm-co-AN) and NTA- PEG-p-(AAm-co-AN). **S5**

Supplementary Fig. 3 The particle size and potential of ES-DSM as E-selectin modifications increased. **S6**

Supplementary Fig. 4 Biocompatibility of blank micelles, DSM and ES-DSM to leukocytes. **S7**

Supplementary Fig. 5 Confocal microscopy images of T lymphocyte and neutrophil 24 hours after the intravenous injection of ES-DSM. **S8**

Supplementary Fig. 6 Flow cytometry detection of 4T1 cells exposed to free Nile red or Nile red-loaded micelles with (+) or without (-) hyperthermia. **S9**

Supplementary Fig. 7 4T1 cell viabilities after exposure to a series of concentrations of blank micelles for 48h. **S10**

Supplementary Fig. 8 Detection of MHC II on DCs after co-incubation with tumor cells. **S11**

Supplementary Fig. 9 Detection of MHC II on DCs after co-incubation with tumor cells in the presence of NECA. **S12**

Supplementary Fig. 10 Analysis of $CD4^{+}$ $Foxp3^{+}$ T cells in the ternary co-incubation system. **S13**

Supplementary Fig. 11 Analysis of $CD4^{+}$ $Foxp3^{+}$ T cells in the ternary co-incubation system containing NECA. **S14**

Supplementary Fig. 12 Average fluorescence intensity of ICG-loaded micelles and ES-modified ICG-loaded micelles in tumor and other major organs 24 h after intravenous injection **S15**

Supplementary Fig. 13 Curves showing the changes of tumor volume of individual mouse after various treatments. **S16**

Supplementary Fig. 14 Microscopic images of H&E-stained cross-sections of the tumors at the end of observation. **S17**

Supplementary Fig. 15 Evaluation of the mature DCs in tumors after different treatments in 4T1 tumor models. **S18**

Supplementary Fig. 16 Evaluation of the mature DCs in sentinel lymph nodes (SLNs) after different treatments in 4T1 tumor models. **S19**

Supplementary Fig. 17 Evaluation of T cells in PBMC and spleen after different treatments in 4T1 tumor models. **S20**

Supplementary Fig. 18 Positive percentage of T cells in tumor calculated based on Fig. 7a and b. **S21**

Supplementary Fig. 19 Immunohistochemistry was used to examine the levels of CD69 and perforin in 4T1 tumor sections. **S22**

Supplementary Fig. 20 The effect of CD39 and anti-CRT antibody on the tumor inhibition of ES-DSM+MW. **S23**

Supplementary Fig. 21 The effect of CD39 and anti-CRT antibody on the tumor infiltration of immune cells. **S24**

Supplementary Fig. 22 Quantitative analysis of bioluminescence signals of mice (n=3). **S25**

Supplementary Fig. 23 Curves showing volumes of rechallenged CT26 tumor of mice after

different treatments (n=6). **S26**
Supplementary Fig. 24 Hemolysis assay to evaluate the biocompatibility. **S27**
Supplementary Fig. 25 Microscopic images of H&E-stained cross-sections of major organs at the end of observation. **S28**
Supplementary Fig. 26 Gating strategies used for cell sorting. **S29**

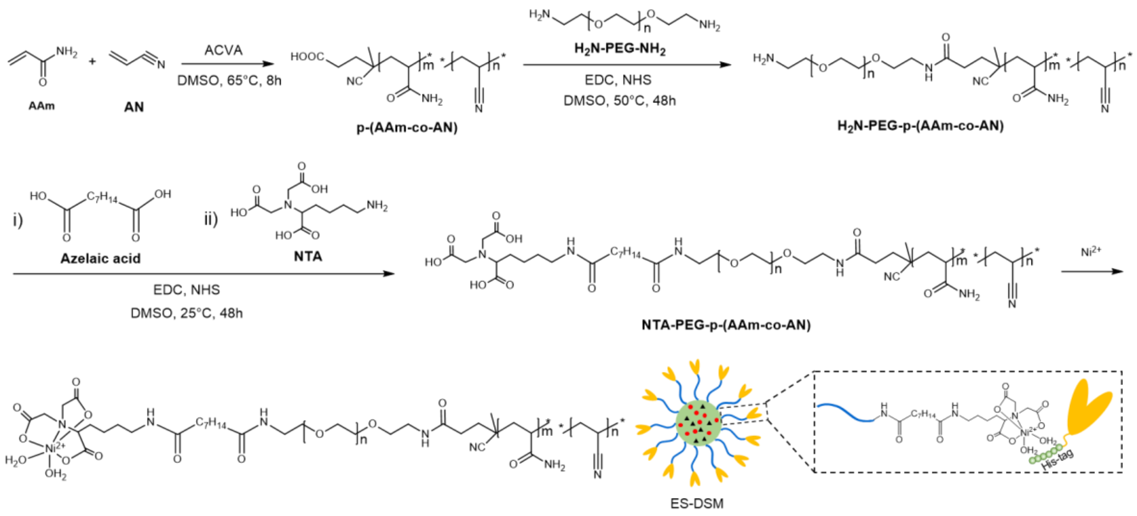

Supplementary Fig. 1 Synthesis route of NTA-PEG-p-(AAm-co-AN) and the illustration of the introduction of E-selectin onto the surface of micelles. The NTA in the polymer was used to chelate Ni^{2+} to afford Ni-NTA, which could further efficiently bind to the His-tag of recombinant E-selectin under a mild condition, thereby introducing E-selectin onto the surface of micelles.

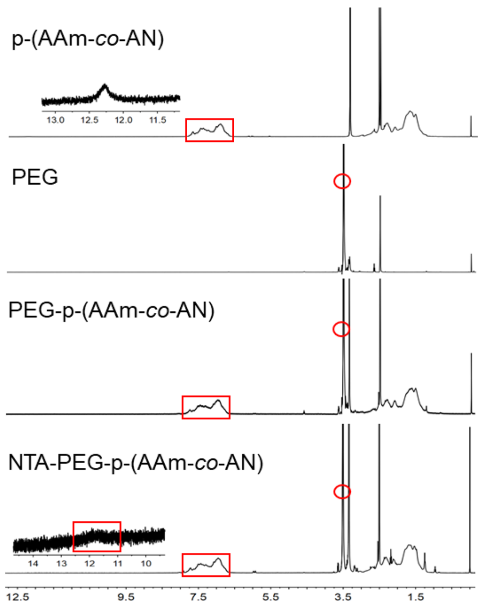

Supplementary Fig. 2 The ^{1}H-NMR spectra of p-(AAm-co-AN), PEG, PEG-p-(AAm-co-AN) and NTA- PEG-p-(AAm-co-AN). The characteristic peak of each polymer was marked. -$CONH_2$ of p-(AAm-co-AN): 6.7-7.9 ppm; -O-CH_2-CH_2- of PEG: 3.6 ppm, -COOH of NTA- PEG-p-(AAm-co-AN): 11.5-12.5 ppm.

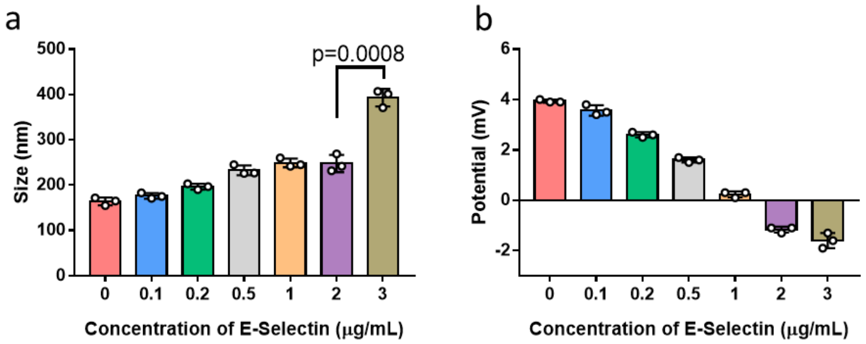

Supplementary Fig. 3 The particle size and potential of ES-DSM as E-selectin modifications increased. **a** The changes of particle size of ES-DSM as the final concentration of E-selectin increased from 0 to 3 μg/mL. When the concentration of E-selectin was 0, the result indicated the size of DSM. **b** The changes of zeta potential of ES-DSM as s the final concentration of E-selectin increased from 0 to 3 μg/mL. When the concentration of E-selectin was 0, the result indicated the potential of DSM. n=3 independent experiments. Data are presented as mean values ± SEM and unpaired two-tailed *T* test was performed in **a**. Source data are provided as a Source Data file.

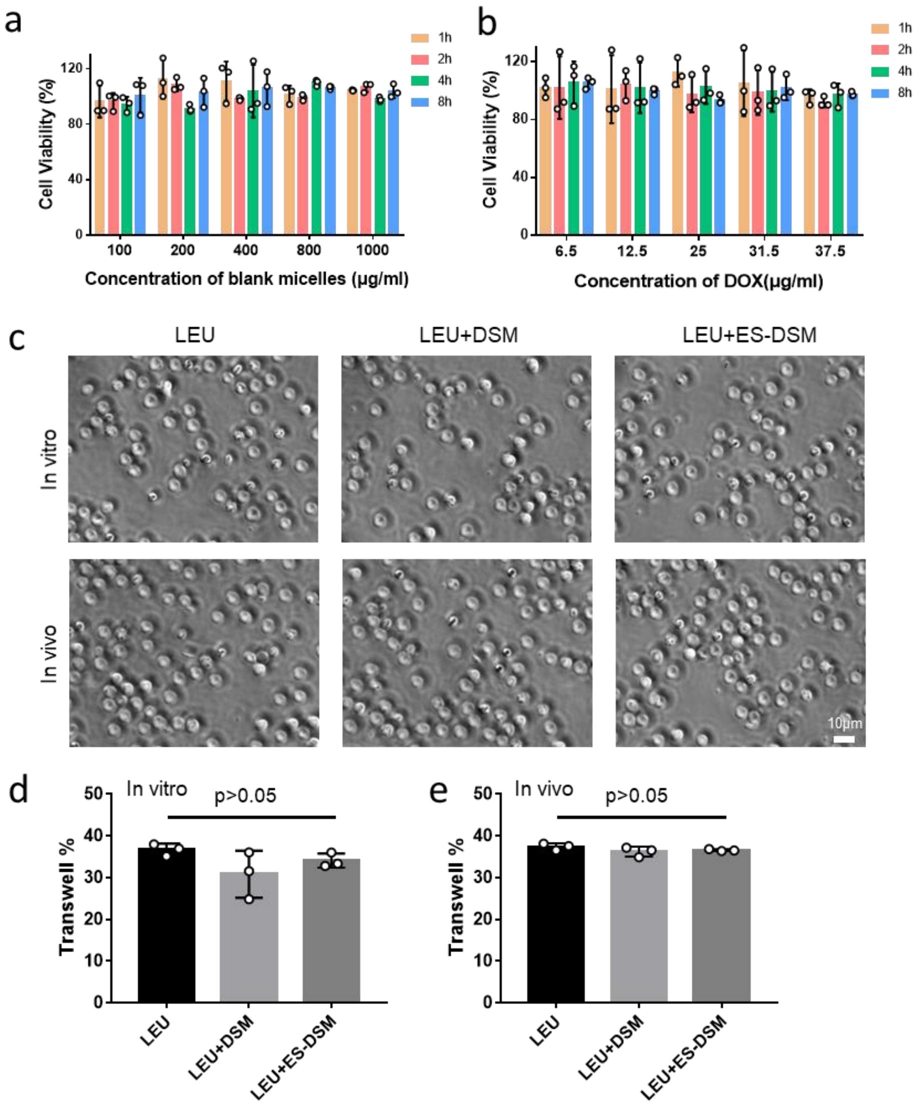

Supplementary Fig. 4 Biocompatibility of blank micelles, DSM and ES-DSM to leukocytes. **a** Leukocyte viability after exposure to a series of concentrations of blank micelles (100, 200, 400, 800, 1000 μg/mL) for different periods (1, 2, 4, 8 h) at 37℃ (n=3 independent experiments). **b** Leukocyte viabilities after exposure to ES-DSM at different DOX concentrations (6.5, 12.5, 25, 31.5, 37.5 μg/mL) for various time (1, 2, 4, 8 h) at 37℃ (n=3 independent experiments). **c** Transwell assay was used to measure the chemotaxis and biological penetration ability of leukocytes (LEU). Images of leukocytes (LEU) transported in the lower chamber of the transwell system in the presence of CXCL2 and CXCL12 were presented. **d** The transwell percentage of LEU after incubation with DSM or ES-DSM in vitro was calculated based on **c** (n=3 independent experiments). **e** The transwell percentage of LEU isolated form mice after intravenous injection of DSM or ES-DSM in vivo was calculated based on **c** (n=3 mice). Data are presented as mean values ± SEM and unpaired two-tailed *T* test was performed in **d**, **e**. Source data are provided as a Source Data file.

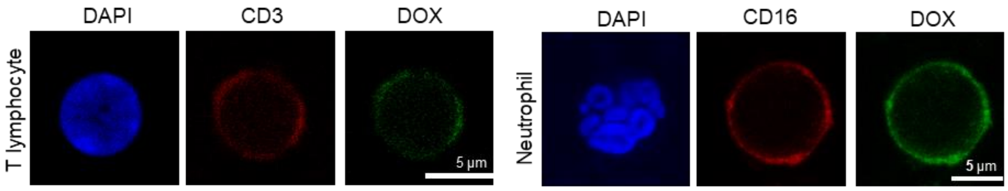

Supplementary Fig. 5 Confocal microscopy images of T lymphocyte and neutrophil 24 hours after the intravenous injection of ES-DSM. Leukocytes were isolated from PBMC and incubated with APC-anti CD3 or CD16 antibody for 20 min at room temperature in dark to identify T lymphocyte and neutrophil, then observed by CLSM. The red fluorescence indicated CD3 on T lymphocyte or CD16 on neutrophil, and the green fluorescence indicated DOX of ES-DSM. The experiments were repeated independently for three times with similar results.

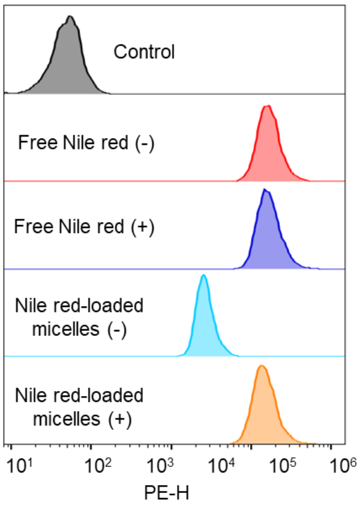

Supplementary Fig. 6 Flow cytometry detection of 4T1 cells exposed to free Nile red or Nile red-loaded micelles with (+) or without (-) hyperthermia. 4T1 cells were exposed to test agents and the hyperthermia treated groups were placed in the cell incubator (43°C and 5% CO_2, 30 min) immediately, followed by incubation at 37°C for 6 h and detected by flow cytometry. Nile red emits strong fluorescence only if it is released from the micelles and binds to cellular lipids. n=2 independent experiments.

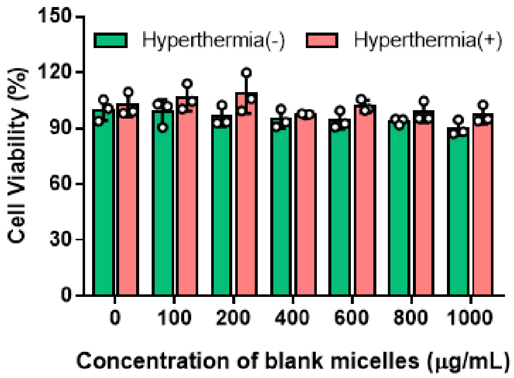

Supplementary Fig. 7 4T1 cell viabilities after exposure to a series of concentrations of blank micelles for 48 h. n=3 independent experiments. Data are presented as mean values ± SEM. Source data are provided as a Source Data file.

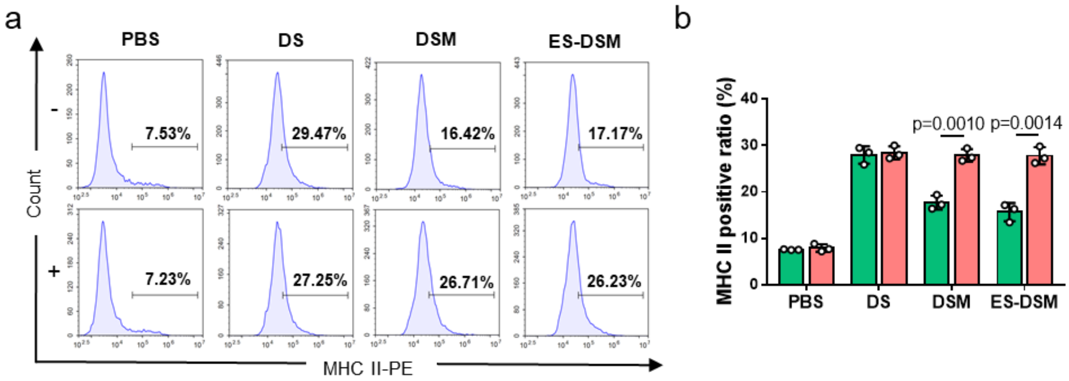

Supplementary Fig. 8 Detection of MHC II on DCs after co-incubation with tumor cells. Tumor cells were pretreated with different agents (PBS, DS, DSM, ES-DSM) with (+) or without (-) hyperthermia, then co-incubated with BMDCs for 48 h. The antigen presenting capacity of DCs was evaluated by the expression level of MHC II. **a** Flow cytometry analysis of MHC II on DCs. **b** Ratios of MHC II positive DCs calculated based on **a** (n=3 independent experiments). Data are presented as mean values ± SEM and unpaired two-tailed *T* test was performed in **b**. Source data are provided as a Source Data file.

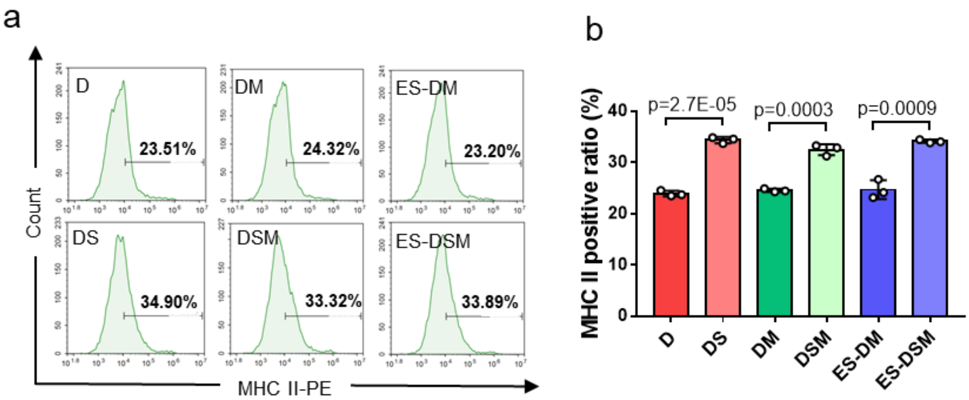

Supplementary Fig. 9 Detection of MHC II on DCs after co-incubation with tumor cells in the presence of NECA. 1 μM of NECA was added in the binary co-incubation system of 4T1 cells and BMDCs, and the immunosuppression relieving effect of SCH was evaluated by detecting the expression level of MHC II on DCs. **a** Flow cytometry analysis of MHC II on DCs. **b** Ratios of MHC II positive DCs calculated based on **a** (n=3 independent experiments). Data are presented as mean values ± SEM and unpaired two-tailed *T* test was performed in **b**. Source data are provided as a Source Data file.

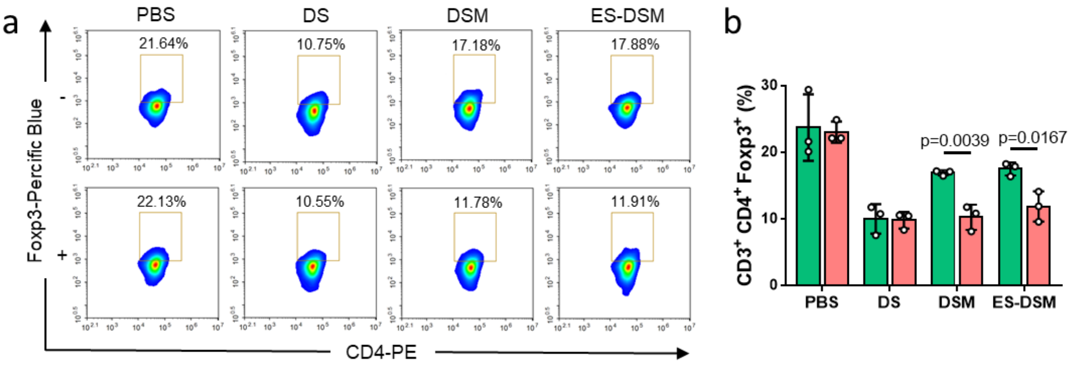

Supplementary Fig. 10 Analysis of $CD4^{+}$ $Foxp3^{+}$ T cells in the ternary co-incubation system. Tumor cells were pretreated with different agents (PBS, DS, DSM, ES-DSM) with (+) or without (-) hyperthermia, then co-incubated with BMDCs and spleen lymphocytes for 48 h. The proportion of Tregs with immunosuppressive effect was detected by flow cytometry. **a** Flow cytometry analysis of the percentages of $CD4^{+}$ $Foxp3^{+}$ T cells after co-incubating with tumor cells and DCs. **b** Ratios of $CD4^{+}$ $Foxp3^{+}$ T cells calculated based on **a** (n=3 independent experiments). Data are presented as mean values ± SEM and unpaired two-tailed *T* test was performed in **b**. Source data are provided as a Source Data file.

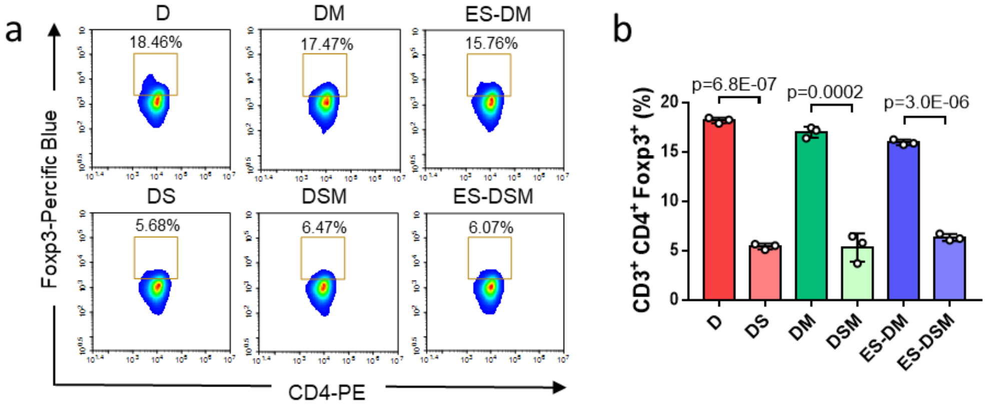

Supplementary Fig. 11 Analysis of $CD4^+$ $Foxp3^+$ T cells in the ternary co-incubation system containing NECA. 1 μM of NECA was added in the ternary co-incubation system of 4T1 cells, BMDCs and spleen lymphocytes, and the immunosuppression relieving effect of SCH was evaluated by detecting the proportion of Tregs. **a** Flow cytometry analysis of the percentages of $CD4^+$ $Foxp3^+$ T cells after co-incubating with tumor cells and DCs with the existence of NECA. **b** Ratios of $CD4^+$ $Foxp3^+$ T cells calculated based on **a** (n=3 independent experiments). Data are presented as mean values ± SEM and unpaired two-tailed *T* test was performed in **b**. Source data are provided as a Source Data file.

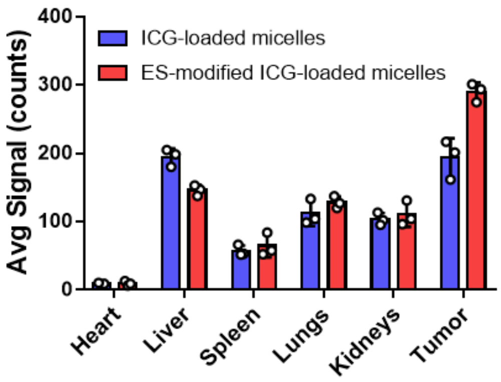

Supplementary Fig. 12 Average fluorescence intensity of ICG-loaded micelles and ES-modified ICG-loaded micelles in tumor and other major organs 24 h after intravenous injection (n=3 mice). Mice were sacrificed and the tissues were isolated, visualized and analyzed by the Maestro in vivo imaging system. Data are presented as mean values ± SEM. Source data are provided as a Source Data file.

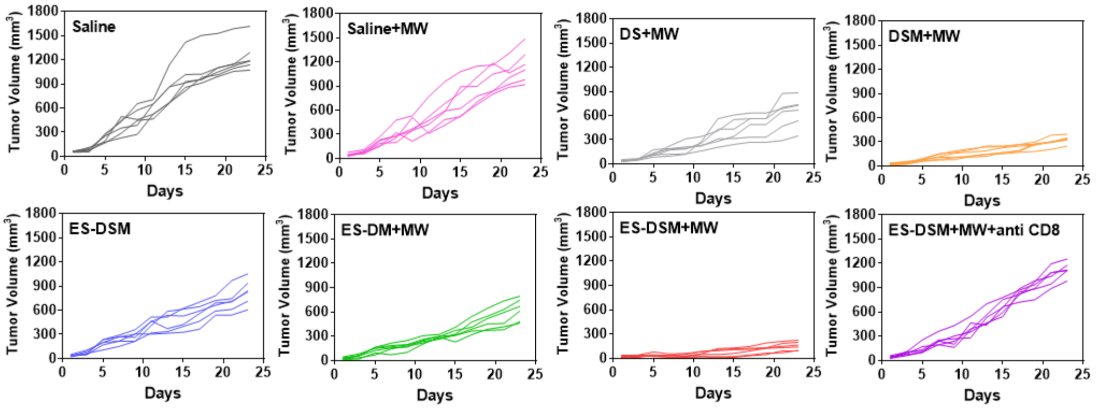

Supplementary Fig. 13 Curves showing the changes of tumor volume of individual mouse after various treatments. Tumor volume of every mice in each group was measured every other day. The mean values and SEM of tumor volume were presented in Fig. 6f. Source data are provided as a Source Data file. n=6 mice.

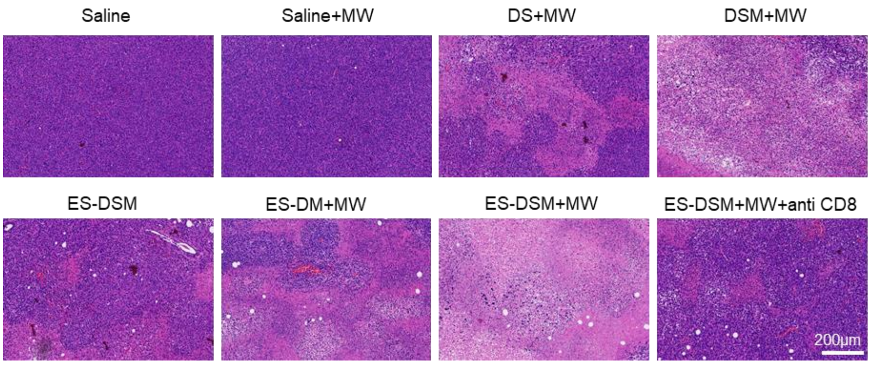

Supplementary Fig. 14 Microscopic images of H&E-stained cross-sections of the tumors at the end of observation. The blue-purple areas represent the nucleus of tumor cells, and the more the area, the more vigorous the tumor proliferation. The pink areas represent the cytoplasm, and the larger the area, the more severe the tumor necrosis. The experiments showed similar results in three independent mice.

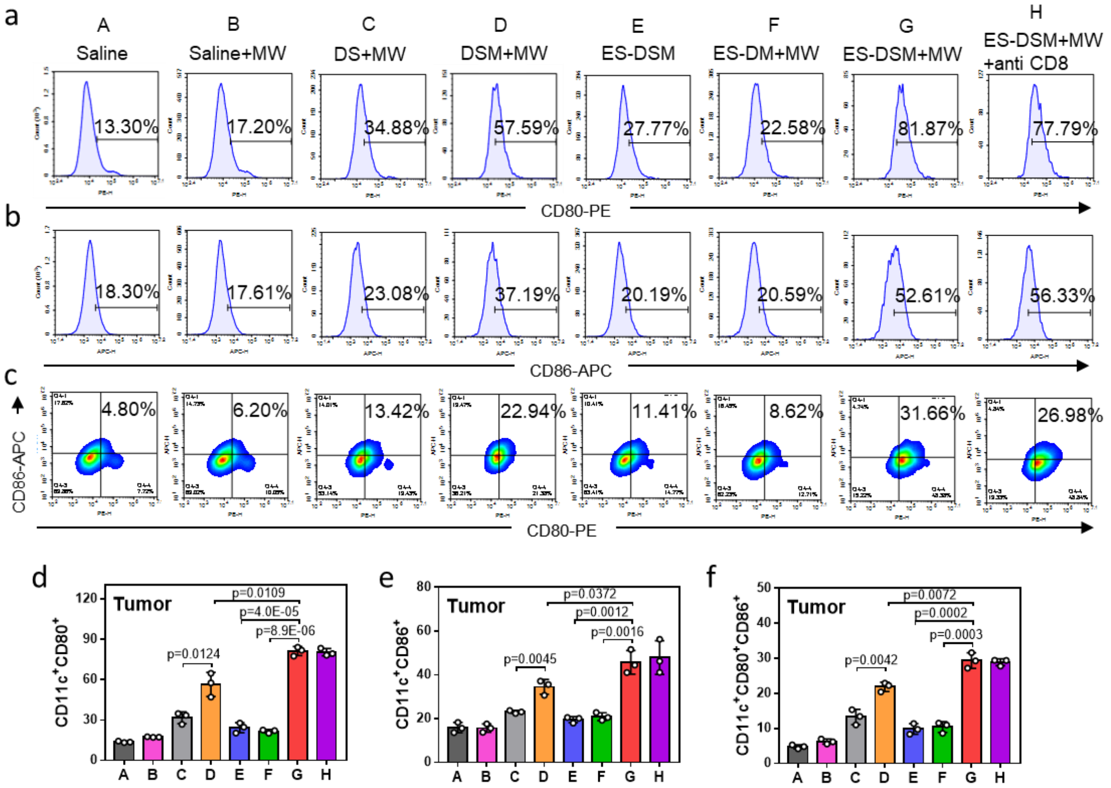

Supplementary Fig. 15 Evaluation of the mature DCs in tumors after different treatments in 4T1 tumor models. At the end of the observation, mice were sacrificed, tumors were isolated and ground through a 200-mesh cell sieve to generate single-cell suspensions. The tumor cells were incubated with FITC-CD11c, PE-CD80 and APC-CD86 antibodies for 20 min at room temperature in dark, analyzed by flow cytometry. The ratios of **a** CD11c^{+}CD80^{+}, **b** CD11c^{+}CD86^{+} and **c** CD11c^{+}CD80^{+}CD86^{+} DCs in tumors of different groups were presented. The percentages of **d** CD11c^{+}CD80^{+}, **e** CD11c^{+}CD80^{+} and **f** CD11c^{+}CD80^{+}CD86^{+} DCs were calculated based on **a**, **b** and **c**, respectively (n=3 mice). Data are presented as mean values ± SEM and unpaired two-tailed *T* test was performed in **d**, **e**, **f**. Source data are provided as a Source Data file.

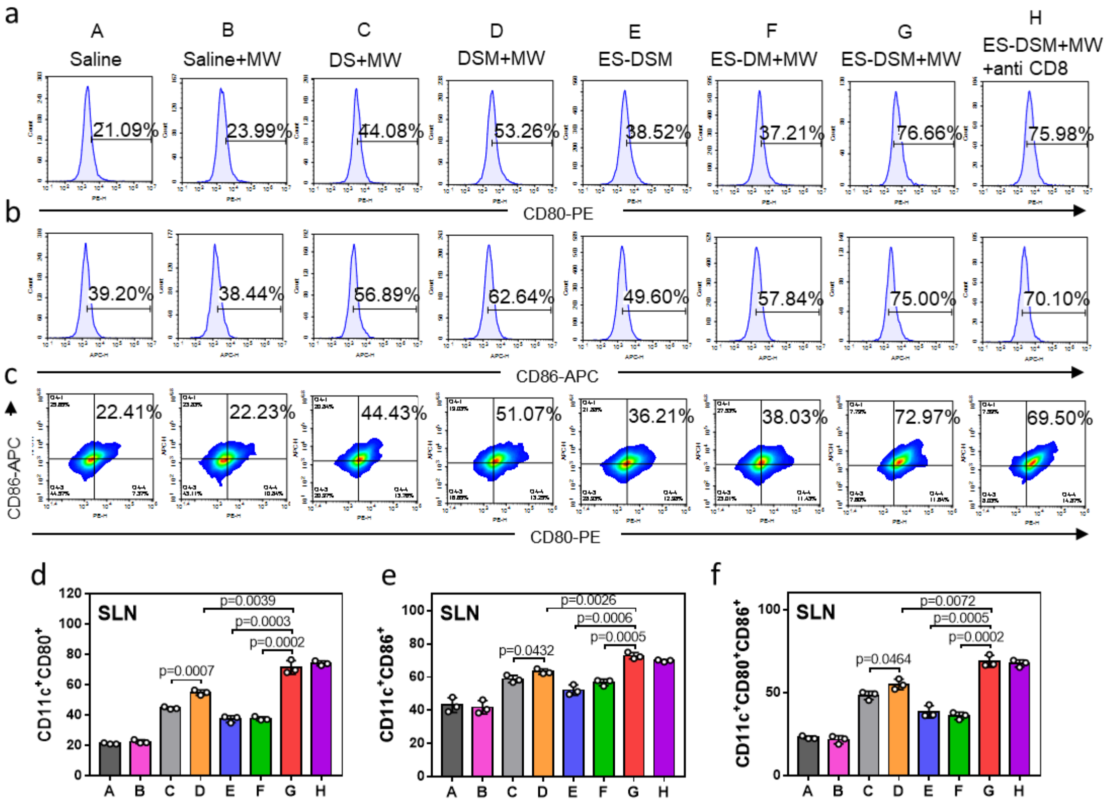

Supplementary Fig. 16 Evaluation of the mature DCs in sentinel lymph nodes (SLNs) after different treatments in 4T1 tumor models. At the end of the observation, mice were sacrificed, SLNs were isolated and ground through a 200-mesh cell sieve to generate single-cell suspensions. The cells of lymph nodes were incubated with FITC-CD11c, PE-CD80 and APC-CD86 antibodies for 20 min at room temperature in dark, analyzed by flow cytometry. The ratios of **a** $CD11c^{+}CD80^{+}$, **b** $CD11c^{+}CD86^{+}$ and **c** $CD11c^{+}CD80^{+}CD86^{+}$ DCs in SLNs of different groups were presented. The percentages of **d** $CD80^{+}$, **e** $CD80^{+}$ and **f** $CD80^{+}CD86^{+}$ DCs were calculated based on **a**, **b** and **c**, respectively (n=3 mice). Data are presented as mean values ± SEM and unpaired two-tailed *T* test was performed in **d**, **e**, **f**. Source data are provided as a Source Data file.

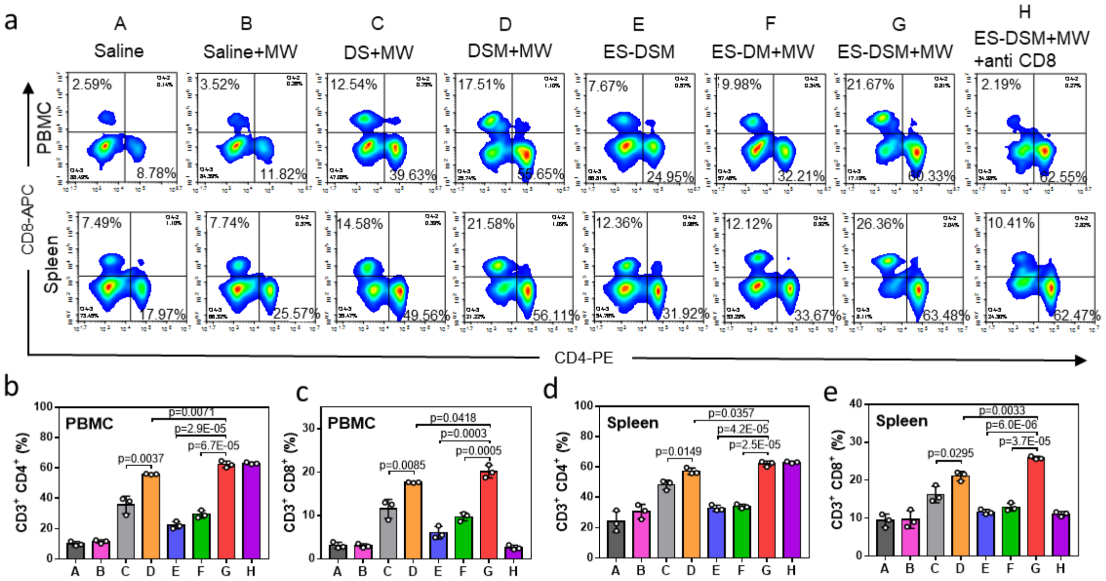

Supplementary Fig. 17 Evaluation of T cells in PBMC and spleen after different treatments in 4T1 tumor models. At the end of the observation, peripheral blood was collected in anticoagulant tubes by enucleation of the eyeball, and PBMC was isolated by density gradient centrifugation. The spleen was isolated after the mice were sacrificed and was ground through a 200-mesh cell sieve to generate single-cell suspensions, the lymphocytes in spleen were isolated by density gradient centrifugation. The PBMC and spleen lymphocytes were incubated with FITC-CD3, PE-CD4 and APC-CD8 antibodies for 20 min at room temperature in dark, analyzed by flow cytometry. **a** Flow cytometry analysis of the ratios of $CD3^{+}CD4^{+}$ and $CD3^{+}CD8^{+}$ T cells in PBMC and spleen lymphocytes. The percentages of **b** $CD3^{+}$ $CD4^{+}$ and **c** $CD3^{+}$ $CD8^{+}$ T cells in PBMC were calculated based on **a** (n=3 mice). The percentages of **d** $CD3^{+}$ $CD4^{+}$ and **e** $CD3^{+}$ $CD8^{+}$ T cells in spleen were calculated based on **a** (n=3 mice). Data are presented as mean values ± SEM and unpaired two-tailed *T* test was performed in **b-e**. Source data are provided as a Source Data file.

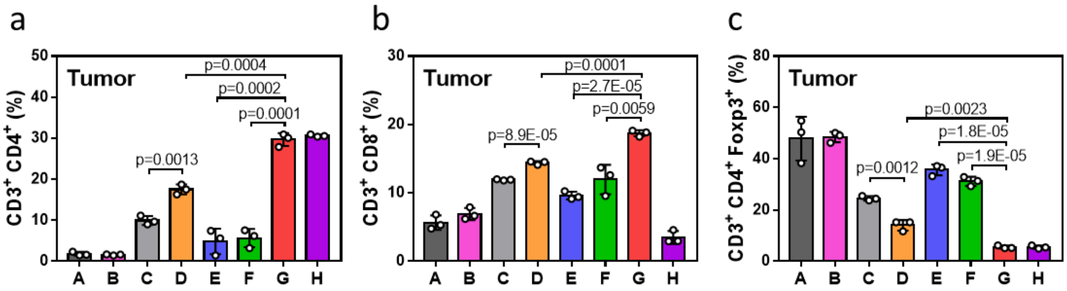

Supplementary Fig. 18 Positive percentage of T cells in tumor calculated based on Fig. 7a and b. The percentages of **a** $CD3^+ CD4^+$, **b** $CD3^+ CD8^+$ and **c** $CD3^+ CD4^+ Foxp3^+$ T cells in tumor (n=3 mice). Data are presented as mean values ± SEM and unpaired two-tailed *T* test was performed in **a-c**. Source data are provided as a Source Data file.

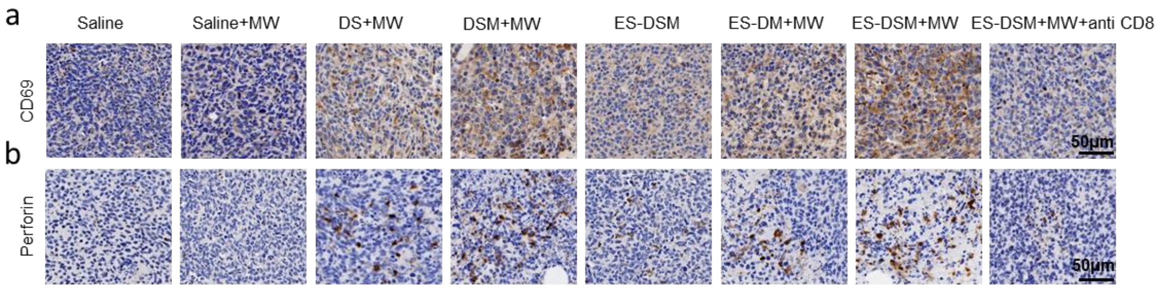

Supplementary Fig. 19 Immunohistochemistry was used to examine the levels of CD69 and perforin in 4T1 tumor sections. The experiments in **a**, **b** showed similar results in three independent mice.

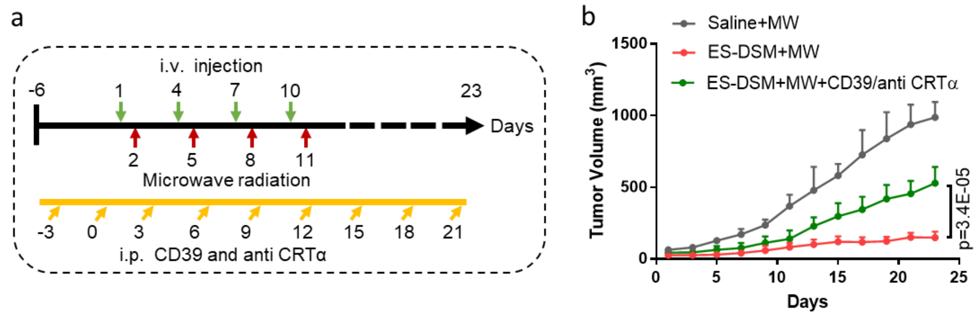

Supplementary Fig. 20 The effect of CD39 and anti-CRT antibody on the tumor inhibition of ES-DSM+MW. **a** Schematic of the treatment regimen. The ES-DSM was i.v. injected on day 1, 4, 7, 10, and the microwave radiation was applied 24 hour later. The CD39 (1 µg/mice per injection) and anti-CRT antibody (10 µg/mice per injection) were injected i.p. every 3 days to deplete ATP and CRT generated during the ICD process. **b** Curves showing tumor volumes of mice after various treatments (n=6 mice). Data are presented as mean values ± SEM and unpaired two-tailed *T* test was performed in **b**. Source data are provided as a Source Data file.

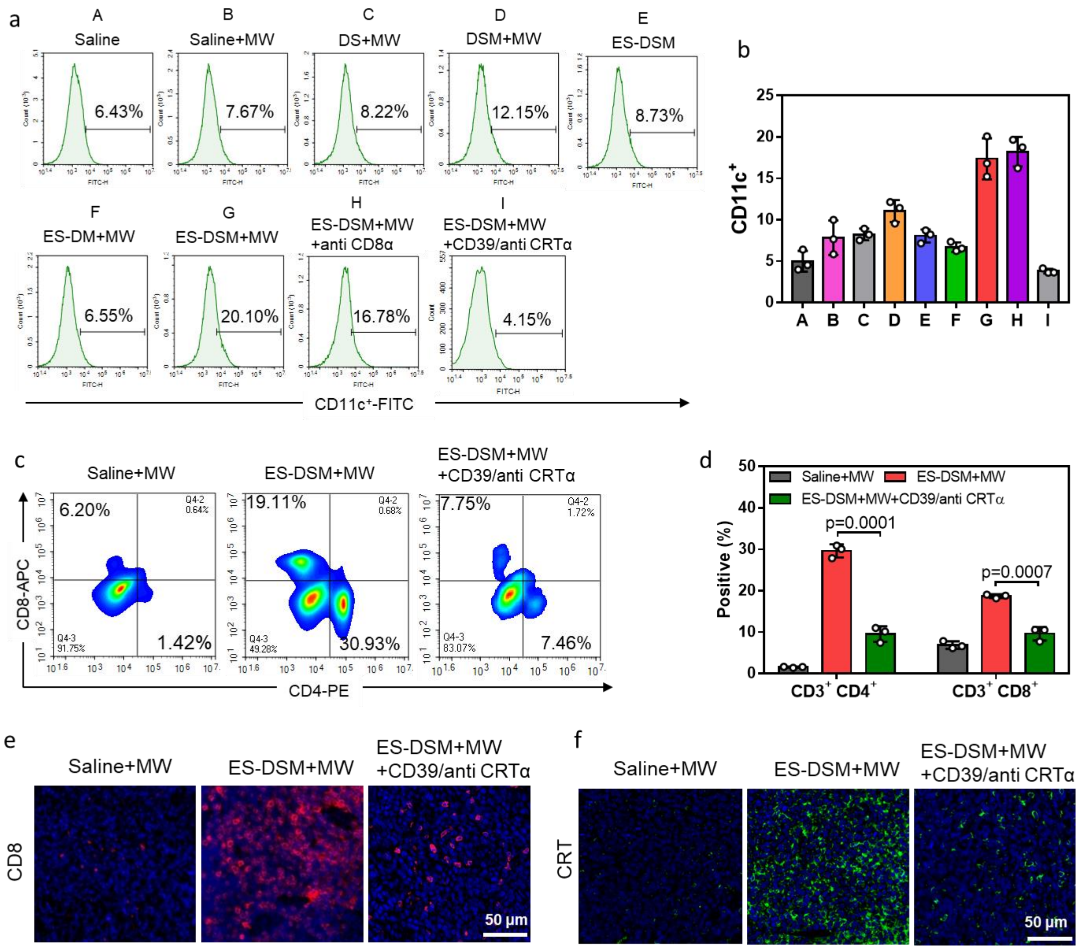

Supplementary Fig. 21 The effect of CD39 and anti-CRT antibody on the tumor infiltration of immune cells. The infiltration of DCs in tumor site is the critical point to elicit antitumor immune response. Tumors were isolated and ground through a 200-mesh cell sieve to generate single-cell suspensions, incubated with FITC-CD11c antibody, or with FITC-CD3, PE-CD4 and APC-CD8 antibodies for 20 min at room temperature in dark, analyzed by flow cytometry. Infiltrations of **a-b** $CD11c^+$ DC and **c-d** $CD3^+CD4^+$ and $CD3^+CD8^+$ T cells in tumors after different treatments were detected by flow cytometry (n=3 mice). Immunofluorescence was used to examine the level of **e** $CD8^+$ T cells and **f** CRT exposure in tumor sections at the end of the observation period. The experiments in **e**, **f** showed similar results in three independent mice. Data are presented as mean values ± SEM and unpaired two-tailed *T* test was performed in **d**. Source data are provided as a Source Data file.

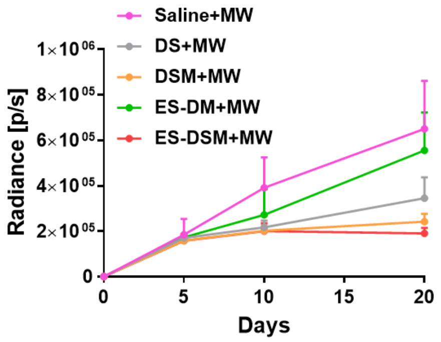

Supplementary Fig. 22 Quantitative analysis of bioluminescence signals of mice (n=3). The radiance signals of pulmonary metastatic tumors of mice in each group were analyzed by IVIS Spectrum imaging system at days 5, 10 and 20, and this result is related to Fig. 8b. Data are presented as mean values ± SEM. Source data are provided as a Source Data file.

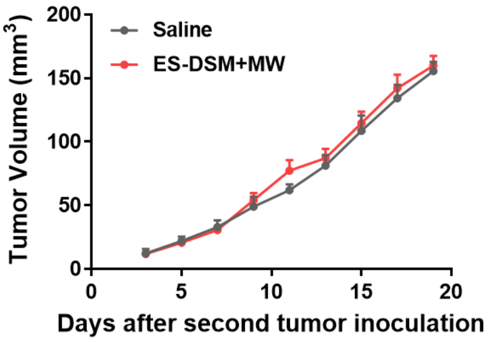

Supplementary Fig. 23 Curves showing volumes of rechallenged CT26 tumor of mice after different treatments (n=6). After orthotopic 4T1 breast tumor-bearing mice were treated with ES-DSM+MW, CT26 cells were inoculated subcutaneously in the left hind limb. The rechallenged CT26 tumor was also monitored every 2 days and the growth curves were presented. Data are presented as mean values ± SEM. Source data are provided as a Source Data file.

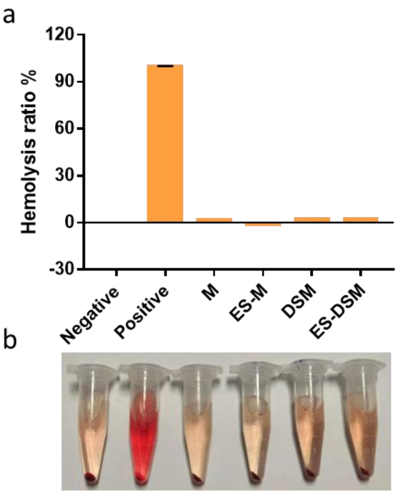

Supplementary Fig. 24 Hemolysis assay to evaluate the biocompatibility. **a** The hemolysis ratios of RBCs after treated with different agents (n=3 independent experiments). **b** The images of the mixture of RBCs and different agents after centrifugation. Source data are provided as a Source Data file.

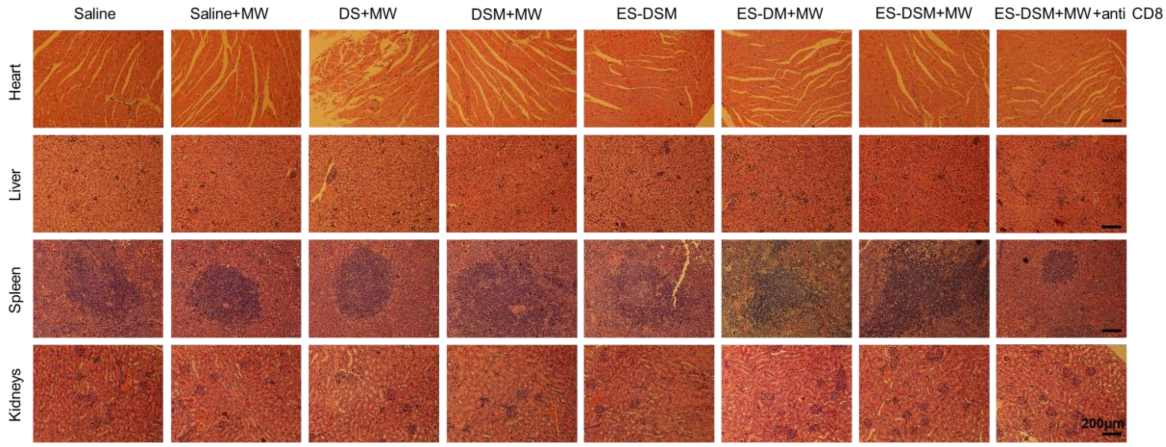

Supplementary Fig. 25 Microscopic images of H&E-stained cross-sections of major organs at the end of observation. The main organs of mice were collected and fixed in 4% paraformaldehyde, embedded in paraffin, cut into 5 μm slices and stained with H&E, then examined under a light microscope. The experiments showed similar results in three independent mice. Scale bar=200 μm.

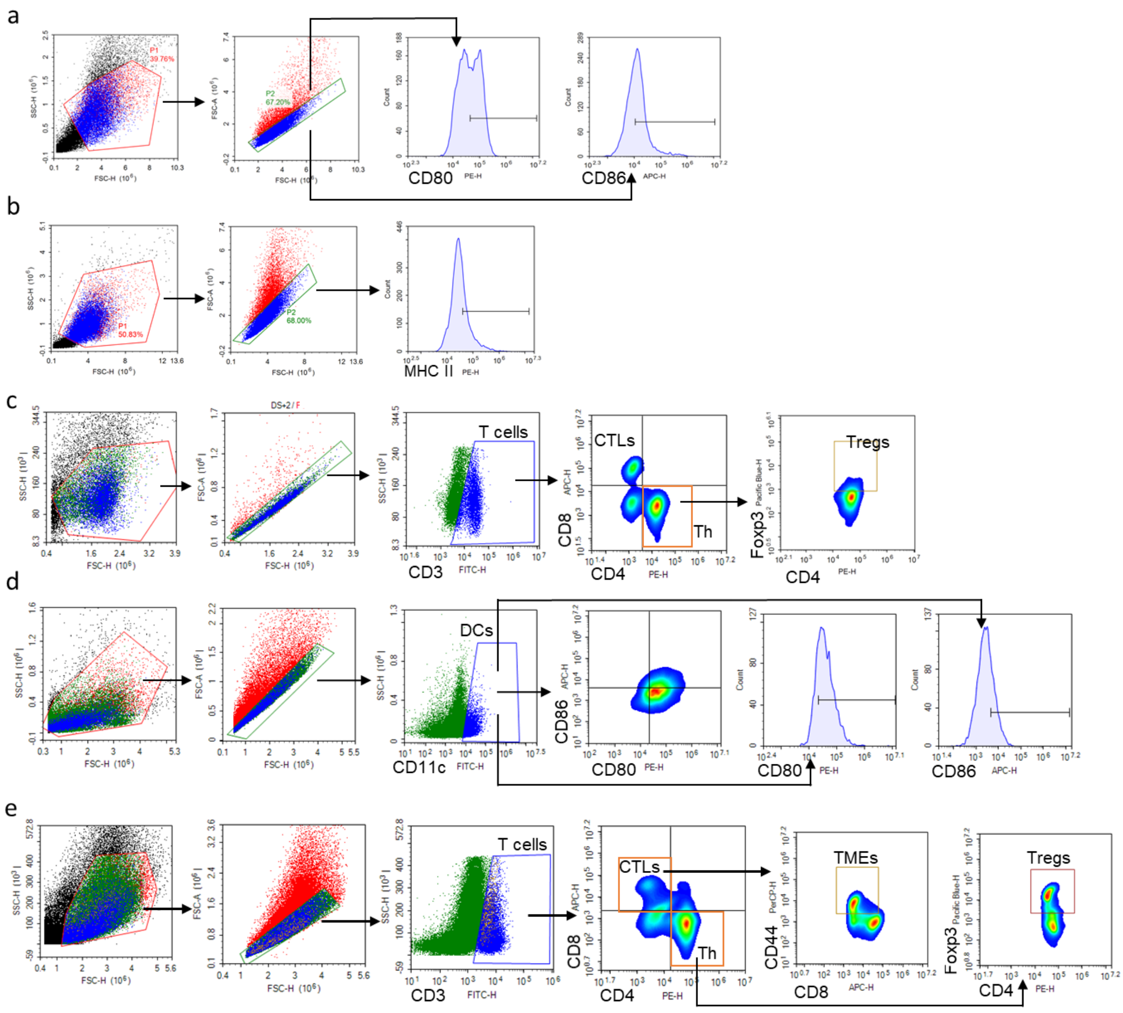

Supplementary Fig. 26 Gating strategies used for cell sorting. **a** Gating strategy to sort mature DCs ($CD80^+$ and $CD86^+$) from the in vitro binary co-incubation system (Fig. 4b, c, d, e). **b** Gating strategy to sort DCs (MHC II^+) from the in vitro binary co-incubation system (Supplementary Fig. 8, 9). **c** Gating strategy to sort CTLs ($CD3^+CD8^+$), Th ($CD3^+CD4^+$), and Tregs ($CD3^+CD4^+Foxp3^+$) from the in vitro ternary co-incubation system (Fig. 5b, c, Supplementary Fig. 10, 11). **d** Gating strategy to sort mature DCs ($CD11c^+CD80^+$, $CD11c^+CD86^+$, and $CD11c^+CD80^+$ $CD86^+$) from sentinel lymph nodes (SLNs) or tumors of 4T1-bearing Balb/c mice (Supplementary Fig. 15a, b, c, Supplementary Fig. 16a, b, c). **e** Gating strategy to sort CTLs ($CD3^+CD8^+$), Th ($CD3^+CD4^+$), TMEs ($CD3^+CD8^+$ $CD44^+$), and Tregs ($CD3^+CD4^+Foxp3^+$) from tumors, PBMC, and spleens of 4T1-bearing Balb/c mice (Fig. 7a, b, c, d, Supplementary Fig. 17a).
